# Gadolinium-Based Contrast Media Nephrotoxicity in Kidney Impairment: The Physio-Pathological Conditions for the Perfect Murder

**DOI:** 10.3390/jcm10020271

**Published:** 2021-01-13

**Authors:** Francesca Martino, Gianpaolo Amici, Mitchell Rosner, Claudio Ronco, Giacomo Novara

**Affiliations:** 1 International Renal Research Institute Vicenza (IRRIV), San Bortolo Hospital, 36100 Vicenza, Italy; cronco@goldnet.it; 2 UO Nephrology, Dialysis and Kidney Transplant, San Bortolo Hospital, 36100 Vicenza, Italy; 3 UO Nephrology and Dialysis, San Daniele del Friuli and Tolmezzo Hospital, ASUFC, 33038 San Daniele del Friuli, Italy; amicig@tin.it; 4 Division of Nephrology, University of Virginia Health System, Charlottesville, VA 22903, USA; mhr9r@hscmil.mcc.virginia.edu; 5 Department of Surgery, Oncology, and Gastroenterology, Urology Clinic University of Padua, 35124 Padova, Italy

**Keywords:** gadolinium-based contrast media, toxicity, kidney damage

## Abstract

Gadolinium-based contrast media (GBCM) toxicity in patients with kidney disease is a concern for the possible development of systemic nephrogenic fibrosis and possible renal complications. This review focuses on the pathological mechanisms underlying the potential kidney toxicity of gadolinium. Gadolinium, as a free compound (Gd3+), is highly toxic in humans because it competes with divalent calcium (Ca2+) and magnesium (Mg2+) ions, interfering in some relevant biologic processes. Its toxicity is blunted by the complexing of Gd3+ with a carrier, allowing its use in magnetic resonance imaging. The binding reaction between gadolinium and a carrier is thermodynamically reversible. Consequently, under some conditions, gadolinium can be released in the interstitial space as a free Gd3+ compound with the possibility of toxicity. Other metals such as iron, copper, and calcium can interfere with the binding between gadolinium and its carrier because they compete for the same binding site. This process is known as transmetallation. In patients with kidney impairment, conditions such as low clearance of the Gd-carrier complex, acid-base derangements, and high serum phosphorous can increase the presence of free Gd3+, leading to a higher risk for toxicity.

## 1. Introduction

Gadolinium is used as a contrast media agent in magnetic resonance imagining (MRI). Until recently, gadolinium-based contrast media (GBCM) was considered to have a low risk of nephrotoxicity or other side effects. However, some reports have demonstrated the potential toxicity of GBCM, especially in patients with kidney impairment [1]. This concern for toxicity was largely related to the association of nephrogenic systemic fibrosis (NSF) in patients with kidney impairment [2] but also with its potential nephrotoxicity [3,4,5]. The pathological mechanisms of NSF have been well described, while a more limited number of reports are available on the mechanisms nephrotoxicity. We hypothesize that gadolinium nephrotoxicity may be underrecognized because it is generally mild and does not affect serum creatinine levels in most cases. Unfortunately, in some conditions such as kidney impairment, a subclinical kidney injury may occur where there is a lack of serum creatinine rise. This injury may decrease kidney reserve and, over time, may manifest as clinically evident chronic kidney disease (CKD).

This review explores the pathological aspects of GBCM toxicity and analyses reports showing how new biomarkers of kidney damage could reveal gadolinium-induced nephrotoxicity.

## 2. The Pathological Mechanism of Gadolinium Toxicity

Gadolinium is a heavy metal of the lanthanide group with a molecular weight of 157 Daltons and known paramagnetic properties. Gadolinium is highly toxic in humans when present as a free ionic compound (Gd3+) [6]. Its toxicity is related to transmetallation processes. Gd3+ has a comparable ionic radius of divalent calcium (Ca2+) and consequently competes with Ca2+ in all biological processes [7]. This competition can lead to an inhibition of voltage-gated calcium channels, with inhibition of nerve impulse transmission and blockage of all Ca2+ dependent enzymes such as de-hydrogenases, kinases, and ATPases [8]. This inhibition may affect mitochondrial function and, more broadly, impair cellular survival.

Furthermore, at physiologic pH, free Gd3+ has a high tendency to precipitate in tissues such as the liver, brain, spleen, kidney, and bone [9,10,11], creating an insoluble salt which activates inflammation and subsequent fibrosis [7,12]. Gadolinium must be bound to a carrier molecule to overcome the toxicity of Gd3+ and to take advantage of its paramagnetic characteristics for body imaging. The reaction between Gd3+ and its carrier is reversible, and the binding force between the carrier and Gd3+, defined by the thermodynamic constant of stability, depends on the type of carrier and external conditions.

### 2.1. The Influence of Carrier Molecules on Complex Stability

In 1988, the first contrast agent specifically designed for MRI, gadopentetate dimeglumine (Magnevist^®^), became available for clinical use. Since then, eight other GBCMs have been developed and approved in many regions worldwide. Currently, GBCM is categorized depending upon their shape (linear versus macrocyclic) and on their charge (ionic versus nonionic). Macro-cyclic carrier molecules bind Gd3+ in a cyclic structure and tend to hold Gd3+ more avidly, while linear carrier molecules tend to have a weaker bond and dissociate from Gd3+ more easily [7,8]. Table 1 reports the principal characteristics of the most commonly used GBCM.

### 2.2. The Influence of External Conditions on Complex Stability

**Figure 1:**
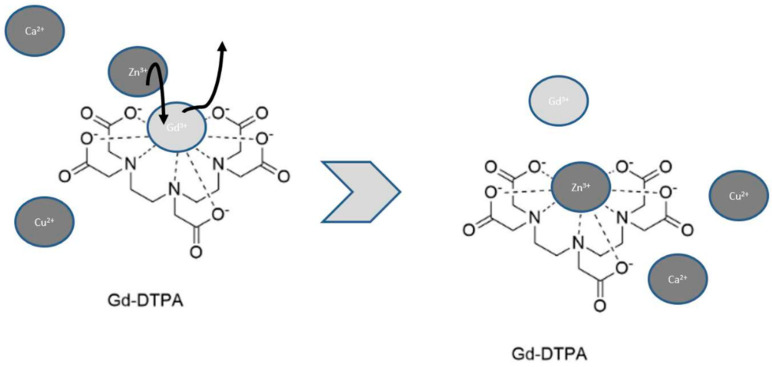
Transmetallation is a dynamic process where the presence of other metals such as Zn2+, Cu2+, and Ca2+ can interfere with Gd3+-carrier complex by competing for the binding site. In this example, Zn2+ displaces Gd3+ with the release of free Gd3+.

The environment plays an important role in the stability of the Gd3+-carrier complex. Specifically, the stability of Gd3+-carrier depends on pH; temperature [13]; the number of free carrier molecules in the solution [14]; and the presence of other metals that can compete for the carrier such as zinc (Zn2+), copper (Cu2+), iron (Fe3+), Ca2+, sodium (Na+), potassium (K+), and magnesium (Mg+) [14]. Cacheris et al. studied how the solubility of the Gd3+-carrier complex was influenced by pH with an acidic pH facilitating the dissociation rate of the Gd3+-carrier complex [15]. Of great importance is that Zn2+, Cu2+, Fe3+, Ca2+, Na+, K+, and Mg+ are competitive with Gd3+ for the binding site of carrier molecules in a process known as transmetallation (Figure 1).

Not surprisingly, different ions show different affinity profiles for a carrier, and these ions also have different endogenous concentrations that affect their ability to compete for binding sites on the carrier. In physiological conditions, the metals that could displace Gd3+ in the transmetallation process are Zn2+, Cu2+, and Ca2+ [12]. In this case, the reaction occurs directly on the Gd3+-carrier complex. Among the endogenous metal ions, Zn2+ seems to have the greatest influence when affecting Gd3+ release from the complex in vivo. As expected, carrier molecules which have a higher selectivity for Gd3+ than for Zn2+ have less toxic complexes with a lower propensity for Gd3+ release. Other endogenous ionic ligands such as citrate, phosphate, and bicarbonate can induce the release of Gd3+ via a different mechanism. These endogenous ligands can form ternary complexes with the Gd3+-carrier complex, and subsequently, the ternary complex dissociates with the release of free Gd3+. Specifically, in the presence of citrate, the dissociation rate of the Gd3+-carrier complex is weak at physiological pH but increases with decreasing pH. Conversely, in the presence of bicarbonate ions, the dissociation rate of the Gd3+-carrier complex increases with higher pH values (7.5–8.5), while in the presence of phosphate ions, the dissociation rate of the Gd3+-carrier complex is higher at pH 6 and lower at pH 8 [13].

**Figure 2:**
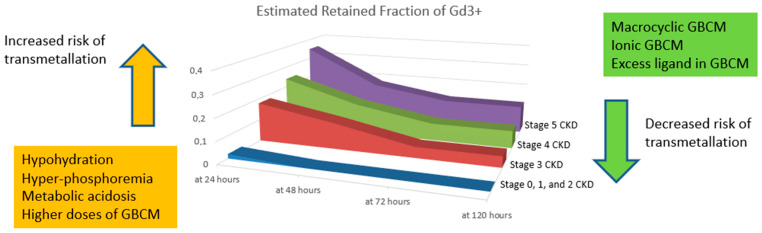
Estimated Gd3+ retention in the simulation model after linear and nonionic GBCM exposure in chronic kidney disease (CKD) patients (modified from [14]): with progressive kidney function impairment, there is an increasing GBCM retainment with higher odds of transmetallation. Furthermore, transmetallation seems to be influenced by other conditions such as the type of GBCM, the GBCM dose exposure, the hydration status, the phosphorus level, the acidosis status, and the fraction of free ligand in GBCM. Specifically, higher doses of GBCM, metabolic acidosis, hyperphosphotemia, and hypoidydration may increase the risk of transmetallation.

In subjects with normal kidney function, the amount of released Gd3+ through the transmetallation process seems to not be clinically relevant, involving about 3.2% of the administered dose, with nearly complete elimination in 48 h. Conversely, in severe kidney impairment, the amount of released Gd3+ is much higher (around 15% of the dose), with only a slight reduction the following day (Figure 2).

This scenario can lead to possible tissue deposition of Gd3+. As stated above, the stability of the Gd3+-carrier complex is affected by pH, being lower at lower pH. Finally, the amount of free carrier plays an important role in the eventual release of Gd3+. The presence of free carriers can allow reuptake of Gd3+ and the formation of new complexes and thus limits toxicity [14]. Finally, the dose and administration route of GBCM are important factors in eventual Gd3+ release. The amount of free Gd3+ is a direct function of the dosage of GBCM, and higher doses are related to a higher likelihood of transmetallation [15]. Specifically, the Royal College of Radiologists’ guidelines suggest avoiding large volumes of GBCM (over 30 mL) to reduce exposure to Gd3+. Furthermore, intra-arterial GBCM administration could theoretically be related to a higher incidence of kidney injury because arterial administration potentially exposes the kidney to a higher concentration of GBCM. Support for this hypothesis can be found in two retrospective studies [16,17], where an incidence of AKI between 11 and 15% was noted with GBCM administered via the arterial route.

## 3. Pathological Mechanism of Gadolinium Nephrotoxicity

The potential nephrotoxicity of Gd3+ seems related both to the physical properties of GBCM such as viscosity and osmolality and to distribution of the Gd3+-carrier complex.

### 3.1. Physical Features: GBCM Viscosity and Osmolality

Osmolality and the viscosity of GBCM have been shown to impact the potential nephrotoxicity [3,18,19]. GBCMs with higher osmolality and lower viscosity than iodinated contrast media (I-CM) have proven to be nephrotoxic [20,21]. GBCMs, similar to I-CM, have higher osmolality and viscosity compared to plasma (normal value 275–295 mOsm/kg [22] and 1.16 to 1.33 Cp, respectively) [23]). In Table 1, we report the chemical-physical characteristics of GBCM.

**Figure 3:**
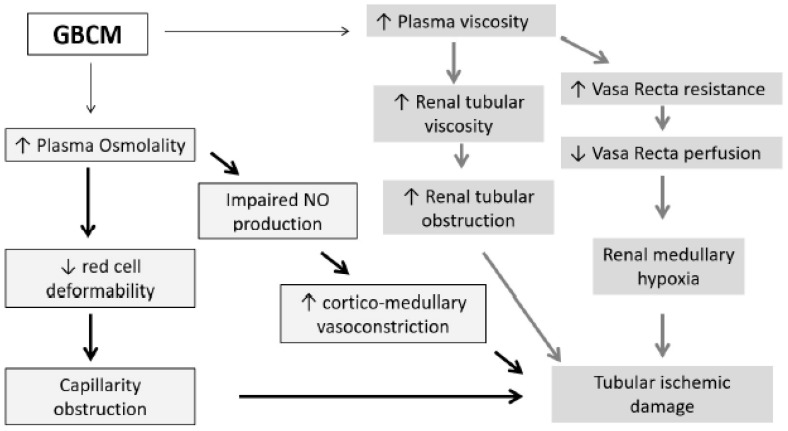
Proposed mechanism of osmolality and viscosity damage in the kidney after GBCM exposure.

The kidney has a unique vascular structure, especially in the vasa recta that can increase vascular resistance. In this setting, the increase in blood viscosity related to the administration of any contrast media has an important impact on blood flow dynamics and can lead to subsequent ischemic damage to the renal tubules (Figure 3) [21].

Furthermore, specifically regarding osmolality, hyperosmotic solutions impair red cell deformability and consequently worsen ischemic damage by leading to capillary obstruction. Finally, the higher osmolality induces protracted vasoconstriction at the cortico-medullary zone by impairment of nitric oxide production [24,25] (Figure 2). These observations have been supported in experimental studies in a porcine model, where exposure to GBCM results in more prominent necrosis of proximal tubules and the presence of protein-filled tubules, a manifestation of proximal tubular functional impairment [20].

### 3.2. Direct Toxicity of Gd3+

Concerning Gd3+-carrier complex distribution, there is some experimental evidence that these complexes concentrate in the kidney tissues, as reported in rat and dog models, where a 5-fold increase in the concentration of the Gd3+ complex in the kidney has been reported [26] compared with concentrations in other organs, such as the lung, heart, and bones. This finding was confirmed in humans by an autopsy performed in a woman with NSF [27]. The higher levels of the Gd3+-carrier complex in the kidney tissues may lead to higher transmetallation and toxicity odds.

### 3.3. Comparison with I-CM

Although the exact mechanisms of kidney damage for GBCM and I-CM are not completely elucidated, both contrast media seem to induce medullary hypoxia and to develop direct toxicity to proximal tubule cells. Theoretically, I-CM damage could be associated with higher viscosity. In contrast, GBCM damage could be related to its higher osmolarity. However, other factors such as the contrast media dose or the hydration status can modulate renal histological and clinical manifestations. Comparing to GBCM, I-CM seems to also have a direct toxic effect on the renal tubular epithelium. Specifically, I-CM tubular toxicity appears related to the stimulation of apoptosis pathways, the disruption of mitochondrial activity, and the perturbation of endoplasmic reticulum activity [28].

## 4. The Role of Chronic Kidney Disease in Gd3+ Toxicity

In chronic kidney disease (CKD) patients, the stability of Gd3+-carrier complexes is altered by changes in kidney function [13,14] for several reasons. Firstly, there is a retention of Gd3+-carrier complexes as their excretion rates fall. Consequently, there is a higher likelihood of Gd3+ release, and consequently, free Gd3+ is available in higher concentrations and may be toxic to tissues. Gd3+-carrier complex retention and Gd3+ release are directly correlated with CKD stage [14], and this aspect partially could explain the higher susceptibility of patients with advanced CKD to kidney damage (Figure 3) as suggested by the clinical data summarized in Table 2.

Secondly, some conditions related to CKD make the kidney a potential target organ for Gd3+ toxicity, such as systemic acidosis and hyperphosphatemia. Metabolic acidosis is a common clinical finding during CKD progression and usually appears when eGFR falls below 40 mL/min. In CKD, there is a progressive decline in ammonium excretion and a concomitant increase in titratable acid in the urine, which primarily uses phosphate as a buffer. However, the relationship between urine pH and CKD remains to be elucidated, although some studies suggested a relationship between urine pH and acidosis status. Specifically, acidosis status (higher renal acid load and lower serum bicarbonate) seems to be associated with the increase of phosphate buffer [13] and the augmented phosphaturia. Thus, there is increased urinary excretion of phosphate in CKD related to increasing parathyroid hormone and acidosis status [52], and FGF23 levels [53]. Theoretically, this condition could lead to the precipitation of Gd3+-PO3—4 salts in the tubular compartment. As proposed in the pathogenetic mechanism of nephrogenic systemic fibrosis, insoluble salts can be phagocytized by macrophages [54,55], with the concomitant activation of inflammatory cells and release of cytokines and other pro-inflammatory mediators. Currently, there is no evidence regarding the possible role of gadolinium phosphate complexes leading to kidney damage [56]. However, histological findings in a human case report [49] showed multifocal acute tubulointerstitial changes with edema and mild mononuclear inflammatory cell interstitial infiltrates, and the tubules with acute injury were focally associated with luminal deposits of calcium-phosphate.

A potentially important mediator of toxicity is NLRP3, a component of a protein complex family related to innate immunity known as inflammasomes. Gd3+ can compete with extracellular calcium in the binding of the calcium-sensing receptor (CSR), which leads to the activation of NLRP3. Active NLRP3 participates in caspase 1 activation, inducing the release of IL1β and IL-18. Specifically, IL1β promotes fibrosis through direct fibroblast stimulation, while IL-18 is related to proximal tubule cell pyroptosis, a catastrophic form of apoptosis and necrosis related to cell lysis [56]. This hypothesis is supported by the significant increase in urine IL-18 concentrations after exposure to GBCM [42].

### Clinical Evidence

Only a few studies documented Gd3+ nephrotoxicity, as reported in Table 2. In these reports, acute kidney injury (AKI) occurred in 12–50% of the cases, with the need for renal replacement therapy in a negligible number of cases.

The development of AKI seems related to prior kidney impairment, the presence of diabetes, exposure to a high dose of GBCM, and the use of linear GBCM complexes. In Gd3+-induced kidney injury, pathological features include the presence of an acute tubulointerstitial injury (characterized by multifocal changes with edema and mild mononuclear inflammatory cell infiltrate in the interstitial space), necrotic tubular cells, and calcium phosphate deposits in tubular lumens. All these findings seem congruent with the findings described in animal models and support the concept of acute tubulointerstitial injury associated with GBCM exposure.

Despite these reports, the infrequent occurrence of AKI after GBCM administration calls into question whether Gd3+ nephrotoxicity occurs or if it is clinically significant at least as measured by changes in serum creatinine, which can be very insensitive to kidney damage. Furthermore, currently, exposure to GBCM is limited in CKD patients with eGFR < 30 mL/min for the concomitant risk of NSF. This circumstance limits, in our perception, the impact of GBCM on the development of AKI in higher risk class patients and, subsequently, the opportunity to better define the risk of AKI in kidney impairment. On the other hand, new biomarkers have emerged in the last ten years to better detect kidney impairment and to allow for earlier detection of tubule damage. For example, N-acetyl-β-D-glucosaminidase (NAG), kidney injury molecule-1 (KIM1), Neutrophil Gelatinase-Associated Lipocalin (NGAL), Interleukin-18 (IL18), insulin-like growth factor binding protein 7 (IGFBP7), and Tissue inhibitor of metalloprotease-2 (TIMP2) have shown their potential utility in the early identification of tubular damage [57]. For Gd3+, the use of some of these biomarkers seems to confirm how GBCM can generate tubular damage. Mawad et al. showed a significant increase in the urine levels of NAG and IL18 3 h after exposure to GBCM [58], while Spasojević-Dimitrijeva et al. demonstrated an increase of KIM1 24 h after GBCM administration [41].

Consequently, kidney damage related to Gd3+ may be subclinical (as defined by no changes in serum creatinine) in most cases and manifests itself when there are other factors such as severe kidney impairment, diabetes, high dose of GBCM, or use of linear GBCM. Currently, no data are available on the consequences of subclinical kidney damage and the prognostic significance of kidney damage biomarker elevations [58]. Despite the lack of evidence about the prognostic value of subclinical kidney damage, we suggest a careful strategy of GBCM administration in patients who have kidney impairment, not only to avoid the risk of AKI but also to preserve the kidney from repeated subclinical damage that may ultimately manifest itself.

## 5. Recommendations for GBCM Use

As reported by most important radiology guidelines, the use of GBCM is currently recommended only when eGFR is over 30 mL/min, while in CKD patients with eGFR under 30 mL/min or in AKI patients, GBCM can be administered only when no alternative examination is available [59,60]. These guidelines for GBCM use may limit the incidence of AKI, but they may not be enough to exclude or avoid Gd3+ subclinical damage to the kidney. Avoiding this damage may be critical in patients who may be exposed to multiple nephrotoxic insults. Clinicians should always assess the opportunities for use of other imaging examinations or the use of MRI without GBCM. When the use of GBCM is absolutely required, macrocyclic ionic compounds at the lowest possible dosage, avoiding the arterial route of administration, should be preferred. Finally, consideration of prophylactic measures such as adequate hydration status, correction of acidosis, and the treatment of hyperphosphatemia should be undertaken. Scientific evidence for the benefit of these prophylactic measures is only available for the administration of fluids. In an experimental study on rats, the administration of fluids before GBCM exposure decreased subsequent rises in serum creatinine [30]. Moreover, in a human study, Takahashi [16] described the possible impact of hydration status on the development of AKI. Finally, in rare cases of stage V CKD where MRI with GBCM is required and no other options are available, an additional hemodialysis session has been suggested to prevent NSF only in those patients who are already treated by hemodialysis. Despite the evidence of the efficient removal of GBCM by hemodialysis treatment with 70% clearance [61,62], no studies have yet investigated the utility of hemodialysis to prevent kidney damage. Peritoneal dialysis may also be effective in GBCM removal as Murashima et al. showed 90% removal of GBMC after two sessions of peritoneal dialysis over the first and second days after GBCM exposure [63].

To summarize, our advice is to use caution with CKD patients (especially when eGFR is under 45 mL/min) and to evaluate strictly the indication of GBCM, weighing the risks and advantages of MRI versus other imaging modalities. When no other imaging options are available, the use of GBCM should be coupled with the use of prophylactic measures as good hydration, avoidance of linear and nonionic GBCM, limitation of the dosage, and perhaps consideration of acidosis and hyperphosphatemia correction. Finally, renal replacement therapy should be suggested only in selective cases.

## 6. Conclusions

GBCMs are usually considered less nephrotoxic than I-CM because they have usually lower viscosity and are generally used at significantly lower volumes. Despite that, Gd3+ can have potential nephrotoxicity especially in the presence of CKD. This may amplify the potential aggressiveness of Gd3+, limiting Gd3+-carrier excretion and promoting the right conditions for transmetallation. The determination as to which conditions can lead to subclinical nephrotoxicity is critical.

## Figures and Tables

**Table 1:** Gadolinium-based contrast media (GBCM) characteristics.

GBCM	Chelant Structure	Charge	Viscosity (mPa’s) at 37 °C	Osmolality (mOsm/kg) at 37 °C	Conditional Stability (logKcond)	Excess Ligand (mmol/L)	Renal Excretion (T1/2 in Hours)	EMA Recommendation
Gadopentetate (Magnevist)	linear	ionic	2.9	1960	18.4	1	1.6	Suspended use in EU
Gadopentetate (Magnevision)	linear	ionic	2.9	1960	not available	not available	not available	Suspended use in EU
Gadodiamide (Omniscan)	linear	non-ionic	1.4	789	14.9	25	1.3	Suspended use in EU
Gadoxetate (primovist)	linear	ionic	1.4	688	18.7	1.3	1.6	Maintained use in EU
Gadoteridol (Prohance)	macrocyclic	non-ionic	1.3	630	17.1	0.5	1.6	Maintained use in EU
Gadobenate dimeglumine (Multihance)	linear	ionic	5.3	1970	18.4	0	1.2–2	Restrict use in liver scan
Gadoversetamide (OptiMark)	linear	non-ionic	2.0	1110	15.0	50	1.7	Suspended use in EU
Gadoterate meglumine (Doratem)	macrocyclic	ionic	2.0	1350	19	0	1.6	Maintained use in EU
Gadobytrol (Gadovist)	macrocyclic	non-ionic	4.9	1603	14.8	1	1.5	Maintained use in EU
Gadoterate (Clariscan)	macrocyclic	ionic	2.1	1350	not available	not available	1.6	Maintained use in EU
Gadoterate (Dotagraf)	macrocyclic	ionic	1.8	1350	not available	not available	1.6	Maintained use in EU
Gadobutrol (Gadovist)	macrocyclic	non-ionic	4.9	1603	14.8	1	1.5	Maintained use in EU

**Table 2:** Preclinical and clinical evidence of GBCM nephrotoxicity.

Report	Study Design	Aim	Number of Subjects	GFR (mL/min)	GBCM	Dose of Gd (mmol/kg)	Results
**Preclinical studies**							
Leader et al. [29]	Experimental animal model	Evaluation of nephrotoxicity in a rabbit model	31	Not reported	Gadopentetate (L, I)	Not reported	Brushborder enzyme (LAP, ALP, and GGT) and lysosomal enzyme of tubular cell increase after GBCM intravenous administration.
Chien et al. [30]	Experimental animal model	Evaluation con 0.9% saline hydration to prevent kidney failure in a rat model	12	Cr-Cl 2.5	Gadodiamide (L, non-I)	5	High doses of GBCM impact kidney function (reduction in Cr-Cl 40%) and lead to vacuolization of proximal tubules. Hydration limits the nephrotoxicity.
Brillet et al. [31]	Experimental animal model	Comparison changing in kidney function between GBMC (M and I) and GBCM (L and I)	GBMC (M, I): 10 GBCM (L, I): 10	Cr-Cl 1.6	Gadoterate (M, I) Gadopentetate (L, I)	Not reported	There is no change in S-Cr with GBCM (M and L); conversely, there is a significant change in S-Cr with GBCM (L and I).
Barbosa Pereira et al. [32]	Experimental animal model	Evaluation of nephrotoxicity in a rat model and acetylcysteine nephron protection	31	16 normal kidney function and 13 with kidney impairment	Gadoterate meglumine (M, I)	Not applicable	In kidney impairment rats, GBCM shows a reduction of GFR. Acetylcysteine seems to reduce nephrotoxicity.
Elmstahl et al. [33]	Experimental animal model	Comparison between GBCM and I-CM group versus control group, intraarterial route	Case group 40 Control group 24	Decutered by nephrectomy	Gadopentetate, (L, I) gadodiamide (L, nonI)	Not applicable	GBCMs are more nephrotoxic than I-CM.
Elmstahl et al. [34]	Experimental animal model	Comparison between GBCM and I-CM, intraarterial route	64	Kidney impaired (Partial nephrectomy)	Gadopentetate (L, I) Gadodiamide (L, non-I)	3 mL/kg	GBCMs induce more kidney damage than I-CM.
Elmstahl et al. [20]	Experimental animal model	Kidney biopsy description	152		Gadopentetate (L, I), Gadobutrol (M, non-I) Gadodiamide (L, non-I)		Necrosis of proximal tubules and glomerulus Hemorrhage and congestion of the cortex, medulla, and glomerulus Vacuolation of proximal tubules Protein-filled tubules in the cortex and medulla
Kwak et al. [35]	Experimental animal model	Comparison of apoptosis in medulla and cortex between the control, GBCM group, and I-CM.	Control:3 GBCM: 9 I-CM: 9	Not reported	Gadopentetate (L, I)	Not reported	No difference in S-Cr between GBCM and I-CM and increase in apoptosis between the control and GBCM
**Clinical studies**							
Safi et al. [36]	Retrospective series	Comparison in the AKI rate between GBCM and I-CM in cirrhotic patients	GBCM: 68 I-CM:84	S-Cr 0.88	Gadobytrol (M, non-I)	Not reported	The rate of AKI (defined as an increase of S-Cr of 0.5 mg/dL) is 17.9% in I-CM and 5.9% in GBCM.
Sambol et al. [17]	Retrospettive series	Comparison between the GBCM group and GBCM + I-CM group	153 GBCM group 59	43.3	Gadodiamide (L, non-I)	Non reported	Rate of AKI (defined as an increase of S-Cr >0.5 mg/dL within 48 h) is 25% in the GBCM group.
Takahashi et al. [16]	Retrospective series	Incidence of AKI after endovascular intervention with GBCM	68	With AKI 18.2 No Aki 25	Gadodiamide (L, non-I) Gadoteridol (M, non-I)	Not reported	The rate of AKI within 48 h is 14.78%. Pre-hydration limits AKI incidence.
Ergun e al. [5]	Retrospective series	Evaluation of CIN after GBCM	91	33	Gadopentetate (L, I), Gadodiamide (L, non-I), or Gadoterate (M, I)	0.2	12% of patients had S-Cr increase (≥0.5 mg/dL)
Briguori et al. [37]	Retrospective series	Comparison after coronary arterial procedure between GBCM and I-CM	GBCM:32 I-CM: 32	Cr-Cl GBCM 33 I-CM 30	Gadobutrol (M, non-I) or Gadodiamide (L-non-I)	<0.4	In the GBCM group, 28% of patients had S-Cr increase (≥0.5 mg/dL), while in the I-CM group, only 6.5% had S-Cr increase (≥0.5 mg/dL).
Sam et al. [38]	Retrospective series	Evaluation of CIN after GBCM	195	38	Gadopentetate (L, I)	0.28	3.5% of patients had S-Cr increase (>1 mg/dL).
Rieger et al. [39]	Prospective series	Evaluation of CIN after GBCM	29	23	Gadopentetate (L, I)	0.34	6.7% of patients had S-Cr increase (≥0.5 mg/dL).
Naito et al. [40]	Randomized trial	Comparison CIN between non-I and I GBCM	102	I: 94.1 Non-I: 90.5	Gadopentetate (L, I) Gadodiamide (L, non-I)	Not reported	Significant S-Cystatin C increase in non-I GBCM
Spasojevic-Dimitrijeva et al. [41]	Prospective series	Comparison kidney damage between I-CM and GBCM	123	133	Gadopentetate (L, I)	0.20	Significant S-Cr and u-KIM1 increase after 24 h
Mawad et al. [42]	prospective series	Evaluation of urinary marker of kidney damage (IL-18, NAG, and NGAL)	28	>60 mL/min	Not reported	Not reported	Significant IL-18 and NAG increase, and no increase in NGAL
Erley et al. [43]	Randomized trial	Comparison of CIN between I-CM and GBCM	21	31	Gadobutrol	0.57	50% of patients had over a 1.5-fold increase in basal S-Cr
Jurgensen et al. [44]	Case report	Description	1	55	Gadoteridol	Not reported	Cr-Cl descreased (<20 mL/min) within 10 days.
Giozzet et al. [45]	Case report	Description	2	80 years: 40 84 years: 23		0.6 0.9	The need for dialysis, and partial recovery of kidney function
Thomsen et al. [46]	Case report	Description	1	20	Gadodiamide (L, non-I)	0.14	Need for dialysis
Schenker et al. [47]	Case report	Description	1	15	Gadodiamide (L, non-I)	Not reported	AKI
Gemery et al. [48]	Case report	Description	1	13	Gadoteridol (M, non-I)	0.44	S-Cr incresed to 9.3 mg/dL.
Akgun et al. [49]	Case report	Biopsy on human	1	1	Gadopentetate (L, I) + Gadodiamide	0.1 + 0.19	S-Cr increased to 3.4 mg/dL. Tubular cell necrosis, tubular cell degeneration, and marked proliferation of tubular cells together with mild interstitial edema and interstitial inflammation
Fujisaki et al. [50]	Case report	Description	1	20	Gadopentetate (L, I)	0.2	Need for dialysis
Badero et al. [51]	Case report	Description	1	38	2 times Gadobenate (L, I) in 24 ore	Not reported. Total volume of 98 cc	S-Cr increased to 7.4 mg/dL

GBCM: Gadolinium-based contrast media, I-CM: iodinated contrast media, L: linear, M: macrocyclic, I: ionic, non-I: Non-ionic, Cr-Cl: Clearance of Creatine, S-Cr: S-Creatine, AKI: acute kidney injury, CIN: contrast-induced nephropathy, u-KIM1: kidney injury molecule-1, IL-18: Interleukin-18, NAG: N-acetyl-β-D-glucosaminidasem, NGAL: Neutrophil Gelatinase-Associated Lipocalin.
